# Therapeutic strategies to recover ependymal barrier after inflammatory damage: relevance for recovering neurogenesis during development

**DOI:** 10.3389/fnins.2023.1204197

**Published:** 2023-06-15

**Authors:** Patricia Paez-Gonzalez, Javier Lopez-de-San-Sebastian, Raquel Ceron-Funez, Antonio J. Jimenez, Luis Manuel Rodríguez-Perez

**Affiliations:** ^1^Department of Cell Biology, Genetics and Physiology, University of Malaga, Málaga, Spain; ^2^Instituto de Investigación Biomédica de Málaga y Plataforma en Nanomedicina-IBIMA Plataforma BIONAND, Málaga, Spain; ^3^Department of Human Physiology, Human Histology, Pathological Anatomy and Sports, University of Malaga, Málaga, Spain

**Keywords:** cell therapy, ependyma, FoxJ1, germinal matrix hemorrhage, neural stem cell, neurogenic niche, neurogenesis, posthemorrhagic hydrocephalus

## Abstract

The epithelium covering the surfaces of the cerebral ventricular system is known as the ependyma, and is essential for maintaining the physical and functional integrity of the central nervous system. Additionally, the ependyma plays an essential role in neurogenesis, neuroinflammatory modulation and neurodegenerative diseases. Ependyma barrier is severely affected by perinatal hemorrhages and infections that cross the blood brain barrier. The recovery and regeneration of ependyma after damage are key to stabilizing neuroinflammatory and neurodegenerative processes that are critical during early postnatal ages. Unfortunately, there are no effective therapies to regenerate this tissue in human patients. Here, the roles of the ependymal barrier in the context of neurogenesis and homeostasis are reviewed, and future research lines for development of actual therapeutic strategies are discussed.

## 1. Introduction

The ependyma is the epithelium covering the surface of the brain ventricles and it is the result of the differentiation and maturation of embryonic neuroepithelium and radial glial cells (Spassky et al., 2005; Malatesta et al., 2008). Ependyma is mainly composed of multiciliated ependymal cells (Spassky et al., 2005; Mirzadeh et al., 2010). During development, neuroepithelium and radial glial cells give rise to all the major cellular components of the brain (Kriegstein and Götz, 2003; Noctor et al., 2008; Kriegstein and Alvarez-Buylla, 2009; Paredes et al., 2016). Neuroepithelium and radial glia act as the neural stem cells in developing brain, they are mono-ciliated and have a long basal prolongation. On the other hand, mature ependyma have lost the stem cell characteristics and the long basal process and have instead differentiated into multiciliated cells (Spassky et al., 2005; Mirzadeh et al., 2010). In healthy conditions, ependyma acts as a barrier against harmful molecules from the CSF (Del Bigio, 1995, 2010; Jiménez et al., 2014; Nelles and Hazrati, 2022), participates in the immune response (Neal et al., 2013), and regulates the CSF circulation between ventricle and parenchyma (Jiménez et al., 2014).

Multiciliated ependyma also supports neurogenesis through direct cell–cell contacts and paracrine signals that generate the appropriate environment to maintain stem cell function (Paez-Gonzalez et al., 2011; Wu et al., 2020) Alteration or disruption of the ependyma causes a disruption of the neurogenic capacity of neural stem cells (Paez-Gonzalez et al., 2011). One of the main causes by which ependyma are disrupted is neuroinflammatory conditions, which can lead to the reversion of ependyma maturity (Abdi et al., 2018).

The last trimester of human development is key for neurogenesis and gliogenesis (Malik et al., 2013). Premature neonates have a high probability of suffering a neuroinflammatory condition. Furthermore, around 25–30% of premature neonates present intraventricular hemorrhages in the germinal matrix (GMH/IVH; Robinson, 2012), and those with severe intraventricular hemorrhages (IVH) will develop posthemorrhagic hydrocephalus (PHH) with severe neurocognitive and sensorimotor problems (Ballabh, 2010). During GMH/IVH, blood cells are released into the ventricular system where they lyse and their neurotoxic components are released into the CSF (Klebe et al., 2015; Tsuji et al., 2020). The hemorrhage produces mechanical pressure on brain tissue, giving rise to cytotoxic edema and necrosis (primary lesion); followed by prolonged neuroinflammation and oxidative stress (secondary lesion) (Klebe et al., 2015; Tsuji et al., 2020). In response to ischemia, microglial cells are activated and secrete proinflammatory cytokines. During the secondary lesion, microglial cells lyse erythrocytes, releasing hemoglobin and iron into the CSF, enhancing the inflammatory response and causing more ependymal damage (Tan et al., 2020; Chen et al., 2021). Inflammation associated with GMH/IVH and PHH entails a serious ependymal cell loss and discontinuities in the ependymal lining (Tsitouras and Sgouros, 2011; McAllister et al., 2017), due to a cascade of events that results in the de-differentiation of ependyma. Ependyma de-differentiation causes a disruption of the microenvironment of the stem cells which impairs neuro- and gliogenesis (Ballabh, 2014; Luo et al., 2019; Flores et al., 2020; Holste et al., 2022). The alteration or disruption of the ependyma causes in addition periventricular edema that increases the previous defects in neurogenesis (Paez-Gonzalez et al., 2011; McAllister et al., 2017).

Current treatments for GMH/IVH and PHH are mostly surgical and directed towards draining cerebrospinal fluid (CSF) to decrease intracranial pressure and do not prevent subsequent complications such as cerebral palsy, sensory deficits, or chronic diseases related to learning and behavior (Romero et al., 2014; Song et al., 2018). These treatments however do not address the neuroinflammation effects on ependyma and the neurogenic niche.

In recent years, new cell-based therapies that take advantage of the regenerative and immunomodulatory properties of stem cells are being attempted (Takahashi et al., 2007; Sadan et al., 2009; Kim et al., 2010; Tang et al., 2017; García-Bonilla et al., 2020). Initially, the designs are based on limited sources of stem cells: human embryonic stem cells (hESC), induced pluripotent stem cells (iPSC) and mesenchymal stem cells (MSC). hESC are derived from human pre-implanted embryos (De Wert and Mummery, 2003), while mesenchymal stem cells can be obtained from less controversial tissues like bone marrow, umbilical cord and placental membranes (Das et al., 2013; Murphy and Atala, 2013). MSC have the benefit of being easily harvested and amplified. Furthermore, iPSC can be obtained from adult skin somatic cells that are reprogramed to a pluripotent state by the induction of the transcription factors Sox2 and Oct3/4 (Bilic and Belmonte, 2012). Additionally, neural stem cells (NCS) can be harvested and isolated from the CSF of infants diagnosed with intracerebroventricular hemorrhage or neural tube defects with therapeutic purpose (Fernández-Muñoz et al., 2020).

Here we review the role of ependyma in controlling, sustaining, and maintaining a normal microenvironment that is capable of sustaining perinatal neurogenesis, and how this control is lost under the inflammatory conditions that follow an GMH/IVH. We then examine how different pathological processes are triggered and highlight the risks of untreated ependymal damage. Finally, we discuss some actual therapeutic strategies currently under development and compare their potential, effectiveness, and gaps.

## 2. Ependyma: final step in the maturation of the neurogenic neuroepithelium

The mammalian central nervous system (CNS) emerges from the neural plate (Stern, 2001; Silbereis et al., 2016), which undergoes the process of neurulation: a thickening of the neural plate and a folding of the tissue at both sides of the dorsal midline, followed by fusion of both neural folds, thus creating the neural tube (Stern, 2001; Echevarría et al., 2003). The neural tube is lined with a pseudostratified germinal neuroepithelium, made up of polarized cells with a single apical cilium and a long basal prolongation that extends and contacts the marginal zone of the CNS (Stern, 2001; Malatesta et al., 2008; Kriegstein and Alvarez-Buylla, 2009; Miranda-Negrón and García-Arrarás, 2022).

**Figure 1:**
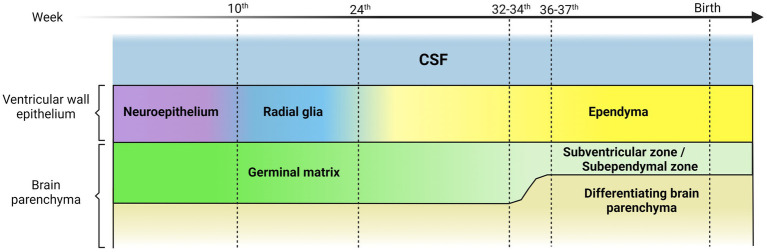
Representative scheme illustrating the transition of the ventricular wall epithelium during human brain development. The ventricular wall epithelium is constituted by different cell types during embryonic development. The first components are the neuroepithelial cells, polarized cells, highly proliferative, with an apical single cilium and a long basal prolongation that extends and contacts the marginal zone. The neuroepithelial cells give rise to radial glial cells with a morphology similar to neuroepithelial cells but with long basal processes that contact the meninges or blood vessels, and their cell bodies are in the ventricular zone. Radial glial cells differentiate mostly into multiciliated ependymal cells. This occurs at around 25 weeks of gestation in humans. Finally, a small subpopulation of radial glial cells differentiates into postnatal stem cells that give rise to neurons and oligodendrocytes. The germinal matrix is situated under and along the ventricular wall epithelium and hosts progenitor and intermediate progenitor cells with high mitotic activity. Neurogenesis and gliogenesis takes place in this region during the embryonic neural development until the 32nd -34 th gestational week in humans, when a decrease in mitotic activity becomes evident. Germinal matrix thickness begins to decrease after the 24 th week of gestation and is practically absent at 36 and 37 weeks of gestation. After the 34 th week, the brain region that is equivalent of the germinal matrix is commonly referred to as germinal ventricular zone or subventricular zone (SVZ) or subependymal zone (SEZ).

During embryonic development, neuroepithelial cells give rise to radial glial cells (Malatesta et al., 2008; Miranda-Negrón and García-Arrarás, 2022; Figure 1). Radial glial cells are a proliferative cell type with a morphology similar to neuroepithelial cells. However, radial glial cells have long basal processes that contact the meninges or blood vessels, and their cell bodies are in the ventricular zone (Angevine et al., 1970; Kriegstein and Götz, 2003; Kriegstein and Alvarez-Buylla, 2009). They give rise to more radial glia through symmetric division, as well as directly or indirectly produce neurons and oligodendrocytes via asymmetric divisions (Haubensak et al., 2004; Noctor et al., 2004; Howard et al., 2008; Noctor et al., 2008).

A subpopulation of radial glial cells differentiates at around 25 weeks of gestation in humans (Sarnat, 1992) into multiciliated ependymal cells, which eventually form the ependymal barrier (Spassky et al., 2005; Merkle and Alvarez-Buylla, 2006; Jacquet et al., 2009; Mirzadeh et al., 2010; Figure 1). Finally, an additional subpopulation of radial glial cells differentiates into neural or postnatal stem cells. These stem cells have their soma below the ependymal layer, in the subventricular zone (SVZ), also called subependymal zone (SEZ) by the position respect to the ependyma (Sarnat, 1992), and they contact the ventricle through a single apical cilium. With the surrounding ependymal cells, they form pinwheel-like, rosette-shaped clusters, which constitute the adult stem cell niches (Mirzadeh et al., 2008).

Interestingly, the postnatal human SVZ/SEZ serves not just as a parenchyma where adult stem cells reside, but as an active tissue which contributes to the ongoing formation and development of the CNS (Tramontin et al., 2003; Beckervordersandforth et al., 2010; Paredes et al., 2016). In fact, it strongly contributes to postnatal neurogenesis for around 2 years after birth, before the remaining neuroblasts start to disappear (Sanai et al., 2011). For this reason, it is essential that the integrity of the ventricular and subventricular region is preserved before and after birth to ensure proper development in humans. The SVZ region is located below ependyma, in the subependymal area. When neurogenesis is finished the SVZ is called subependyma (SE). This transformation occurs at different moments of the development in the different regions of the ventricular walls (Puelles, 2021).

## 3. Ependyma is directly affected by GMH/IVH and PHH

Intraventricular hemorrhages affect 20% of premature neonates (Ballabh, 2010) mainly originated by a failure of the vasculature located in the germinal matrix (Volpe, 1989; Hansen et al., 1998; Ballabh, 2014).

The germinal matrix hosts an exceptional number of progenitor and intermediate progenitor cells (Sanai et al., 2011; Ballabh, 2014; Paredes et al., 2022) contains enormous mitotic activity related to stem cells and progenitor cells (Tramontin et al., 2003), and most of neurogenesis and gliogenesis during the embryonic neural development occurs in this region (Doetsch et al., 1997; Tsitouras and Sgouros, 2011; Leijser and De Vries, 2019), until the 32nd -34th week of gestation time in humans (Sanai et al., 2011). Germinal matrix thickness begins to decrease after the 24th week of gestation and is nearly absent by 37 weeks of gestation (Gould and Howard, 1988; Ballabh et al., 2004; Del Bigio, 2010; Strahle et al., 2012; Ballabh, 2014). After 34 weeks, the germinal matrix becomes commonly referred to as the germinal ventricular zone (Nadarajah and Parnavelas, 2002; Chen et al., 2018; Luo et al., 2019). Consequently, germinal matrix integrity remains chiefly important, especially in preterm infants (Luo et al., 2019; Figure 1).

**Figure 2:**
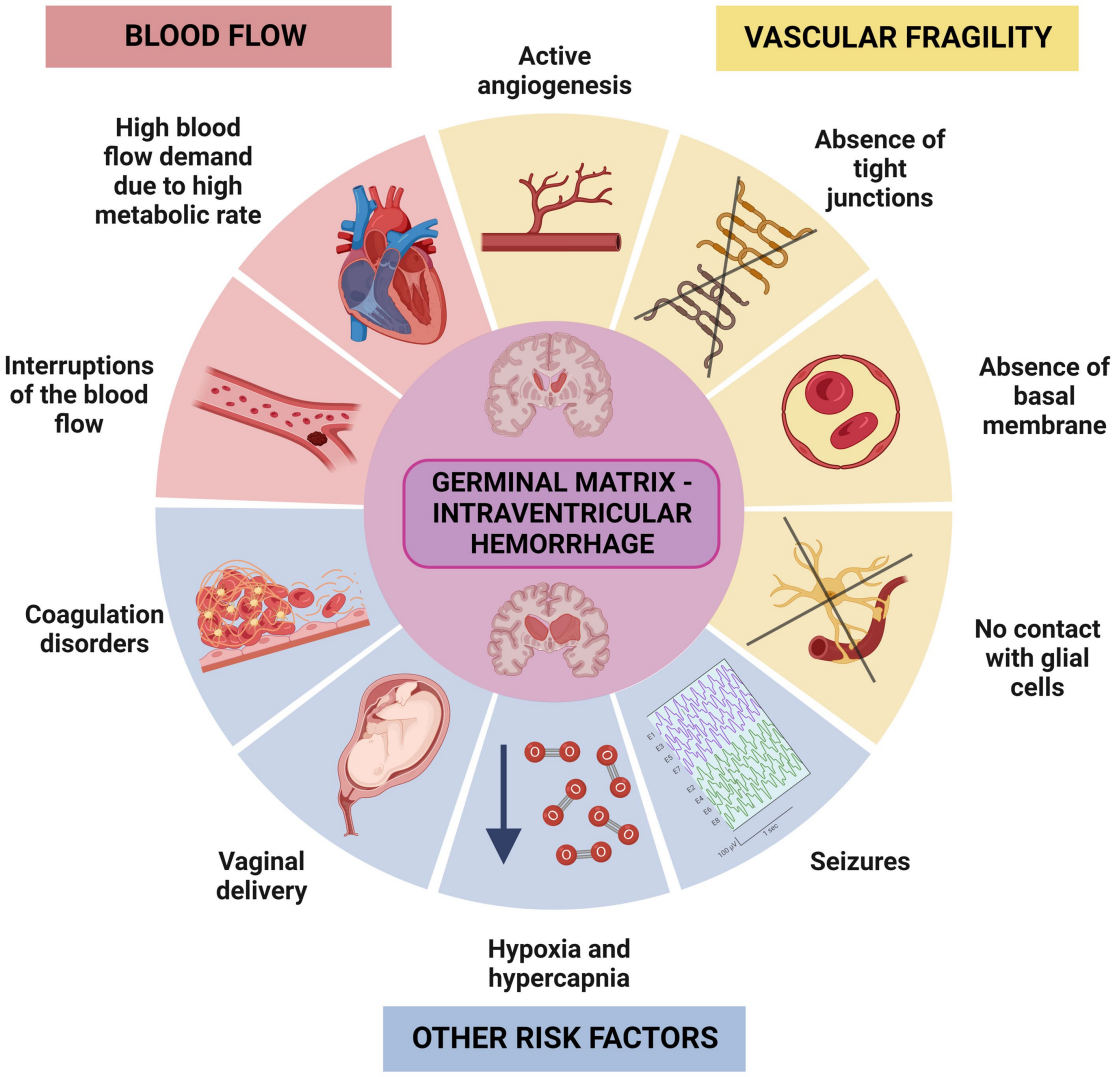
Representative scheme illustrating the factors that can influence the IVH in the germinal matrix. There are two main group of risk factors that increase the probability of suffering IVH in the germinal matrix, one related to fragility of the blood vessels, and another related to increased demand for blood flow. Hypoxia, seizures, coagulation disorders or problems during the vaginal delivery are also risk factors that may trigger IVH.

The germinal matrix is the brain region most susceptible to hemorrhaging during brain development. The germinal matrix hemorrhages (GMH) mainly appear between the ventricular wall and the caudate nucleus within the caudothalamic groove, and they may progress into the brain ventricles causing intraventricular hemorrhage (Volpe, 1989; Hansen et al., 1998; Ballabh, 2014). Several risk factors have been described that increase the probability of suffering GMH in the germinal matrix, such as vascular fragility (Ballabh et al., 2004; Ballabh, 2014; Luo et al., 2019). During angiogenesis, the blood vessels typically have a weak basal membrane, lack tight junctions, and are not yet in contact with glial cells. Despite this, they receive an enormous amount of blood flow to maintain the high metabolic demand of the region (du Plessis 2008, 2009; Ballabh, 2014). Additionally, interruptions of cerebral blood flow, and coagulation and platelet disorders have been demonstrated to cause GMH/IVH (Kenny et al., 1978; Volpe, 1989; DiSalvo 1998). Other risk factors include vaginal delivery, hypoxia, hypercapnia, and seizures (Kenny et al., 1978; Volpe, 1989; DiSalvo 1998; Figure 2). Depending on the severity of the hemorrhages, both neurogenesis and gliogenesis can be critically impaired leading to serious sequelae (Leijser and de Vries, 2019; Luo et al., 2019; Flores et al., 2020). The location of the germinal matrix with respect to the ventricular wall epithelium is key to understanding how the hemorrhages that occur in the germinal matrix may affect to the integrity and functionality of the ependyma. The germinal matrix is situated under and along the ventricular wall epithelium (Tramontin et al., 2003). Depending on the number of weeks into brain development, germinal matrix will be located under the proliferative neuroepithelium or under the immature ependyma (Tramontin et al., 2003; Figure 1).

**Figure 3:**
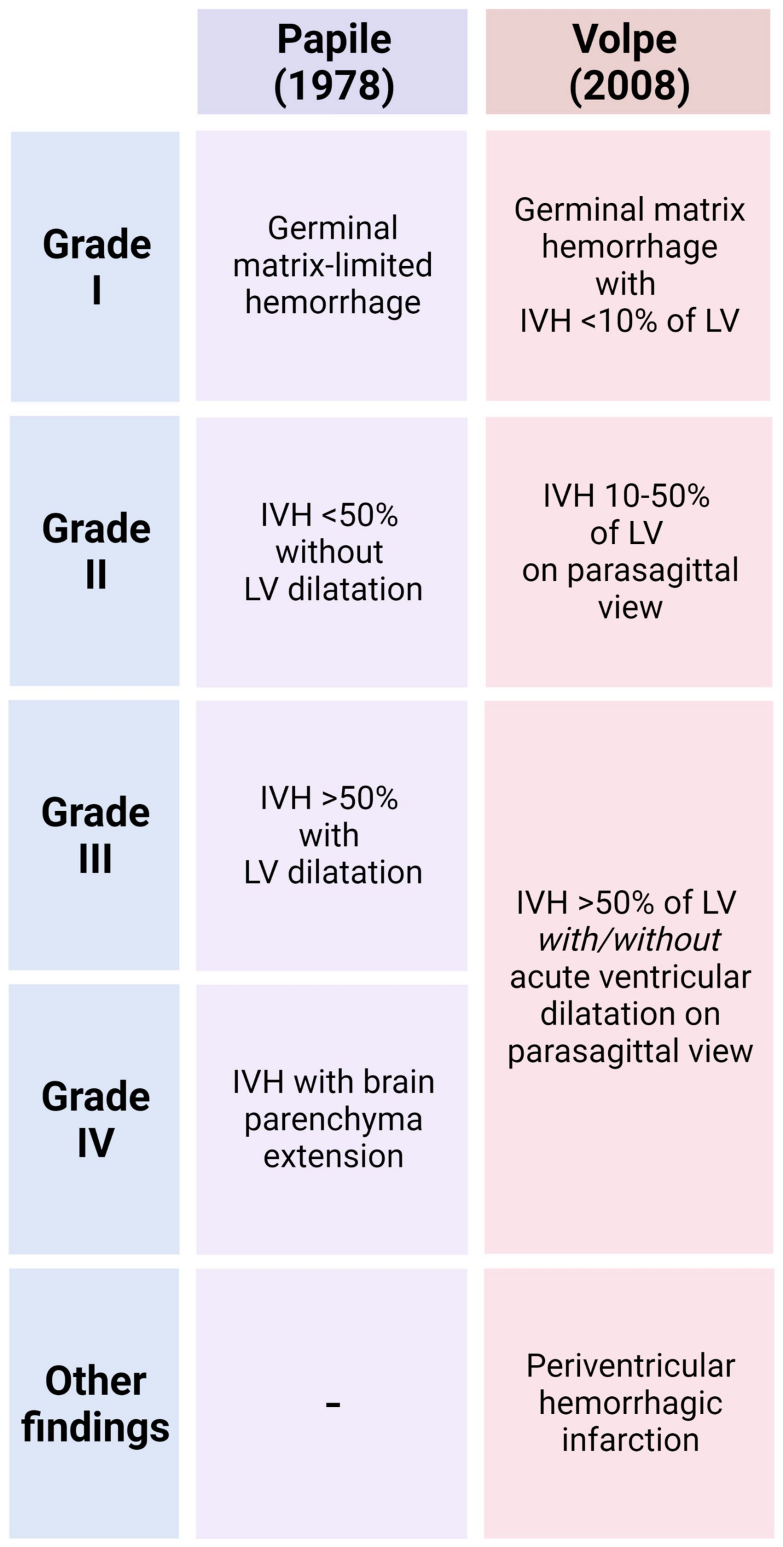
Main characteristics that define of GMH/IVH in the Papile System and Volpe System. For each grade, the extent of the hemorrhage and the percentage of blood detected into the ventricle cavities that allows the classification is indicated.

**Figure 4:**
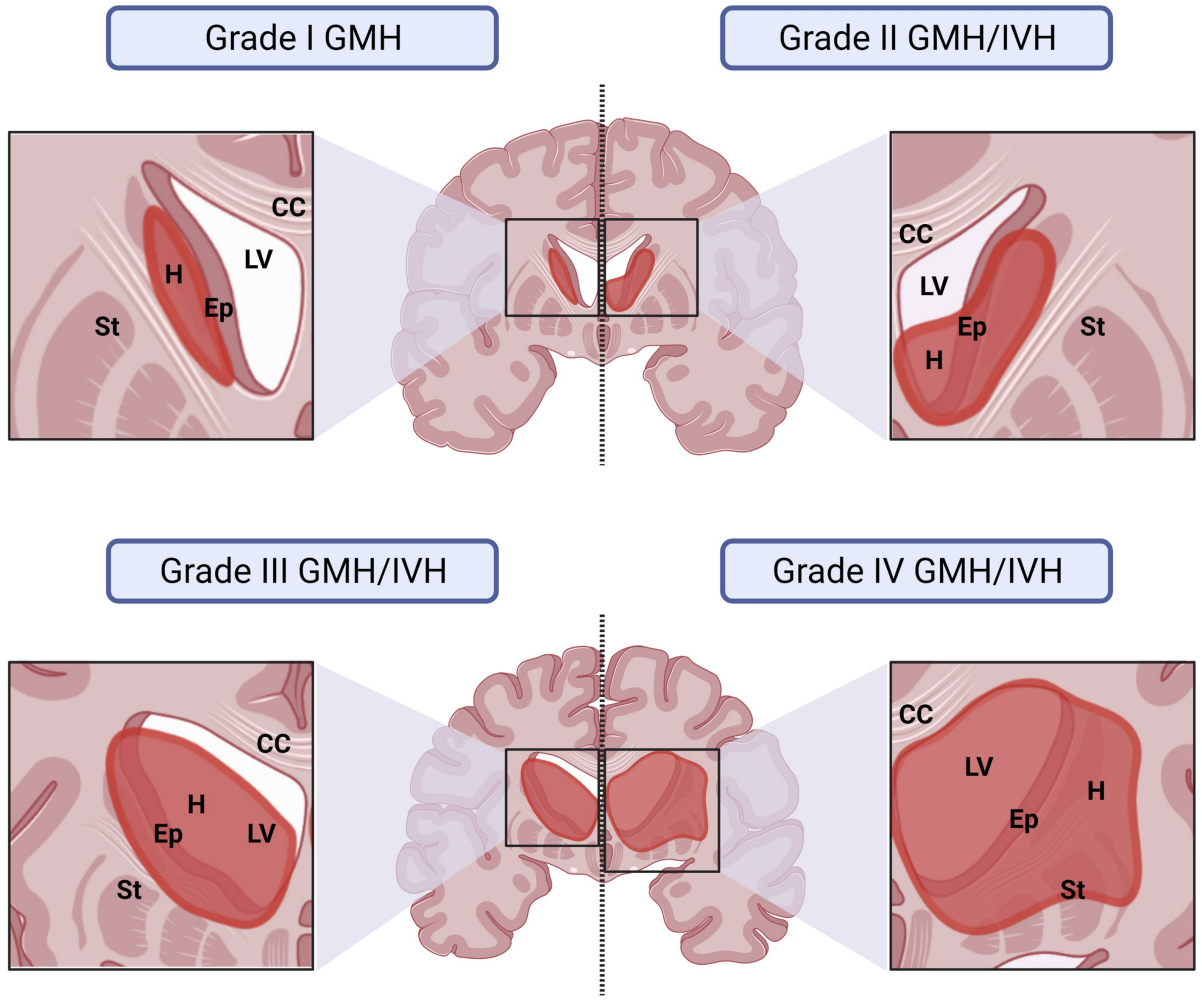
Representative drawings illustrating how the germinal matrix IVH affects the surrounding structures depending on the grade of severity of the IVH. The IVH has been graded under the Papile system. In grade I, hemorrhage affects only to the germinal matrix. In this case, IVH will affect to the ependyma if the hemorrhage occurs close to the ventricular border. The damage is usually minimal. In grade II, the hemorrhage occurs in the germinal matrix but progresses into the ventricles, however, IVH does not cause ventricular dilatation. Ependyma is damaged and blood products spread into the CSF affecting more ependymal areas. In grade III the hemorrhage that occurs in the germinal matrix progresses into the ventricles and, additionally, causes ventricular dilatation. It will be also considered grade III if the IVH occupies more than 50% of the ventricular cavity, even if no ventricle dilation is detected. The damage in the ependyma in this grade is severe, the blood products reach more areas of the ventricle cavities and blood by-products induce a more generalized inflammatory response. In grade IV the hemorrhage has spread to the brain parenchyma and the ependyma disruption and inflammatory response will be severe. CC, Corpus Callosum; Ep, Ependyma; GMH, Germinal Matrix Hemorrhage; H, Hemorrhage: IVH, Intraventricular Hemorrhage; LV, Lateral Ventricle; St, Striatum.

Due to the close proximity of the germinal matrix and the ependyma, depending on the severity of the IVH, the ependyma will be affected at different degrees (Papile et al., 1978; Volpe, 1989; Volpe et al., 2017). The severity of GMH/IVH has been graded to unify diagnosis and treatments. There are two different systems commonly used to grade GMH/IVH. In the Papile system, hemorrhages are classified from Grade I to IV (Papile et al., 1978; Figure 3). In Grade I, hemorrhage appears only in the germinal matrix; in Grade II the hemorrhage progresses into the ventricles without causing ventricular dilatation; in Grade III the hemorrhage progresses into the ventricles causing ventricular dilatation and/or the hemorrhage occupy more than 50% of the ventricle cavity; and finally, in Grade IV the hemorrhage has spread to the brain parenchyma (Figure 3). In the Volpe system, Volpe eliminates Grade IV of the GMH/IVH, as it is considered another type of disease (Volpe et al., 2017; Figure 3). Profuse bleeding will reach the ependyma and fill the ventricle with blood (Papile et al., 1978; Volpe, 1989; Volpe et al., 2017; Figure 4). The presence of blood and the presence of inflammatory molecules and cells during and after the GMH/IVH (Holste et al., 2022) constitute a serious threat to ependymal integrity. The components and by-products contained in the blood may hinder ependymal cells function and survival (Dawes, 2022).

Following the GMH/IVH, preterm/neonates that suffer from germinal matrix hemorrhage can develop post-hemorrhagic hydrocephalus (PHH). In case of severe GMH/IVH, 75% of premature neonates will develop PHH (Ballabh, 2010). PHH was traditionally believed to be caused by cerebrospinal fluid (CSF) accumulation in the brain ventricles due to a flow obstruction, such as an occlusion or obstruction of the ventricular aqueduct or the outflow tracts (Karimy et al., 2020). However, CSF can also accumulate due to CSF hypersecretion caused by an inflammation dependent process that favors choroidal plexus sodium-potassium-chloride cotransporter NKCC1 function (Lolansen et al., 2022). CSF hypersecretion is mediated by Toll-like receptor 4 (TLR4), a surface receptor protein and NF-κB pathway activator (Karimy et al., 2017) that is linked to the inflammatory response of the tissue after GMH/IVH (Toft-Bertelsen et al., 2022). Additionally, TLR4 can be activated through the serum lipid lysophosphatidic acid (LPA) agonistic action over the transient receptor vanilloid 4 cation channel (TRPV4) activation (Toft-Bertelsen et al., 2022).

When GMH/IVH evolves into PHH, ependymal damage could be considered to be both a cause and a consequence of the PHH. The initial loss of ependyma due to GMH/IVH does impair CSF flow, leading to the onset of hydrocephalus, which itself exerts further damage to the ependyma (Sevensky et al., 2021). Altogether it entails a serious ependymal cell loss, discontinuities in the ependymal lining and disruption of the subventricular tissue (Tsitouras and Sgouros, 2011; McAllister et al., 2017) and neurogenic niche.

## 4. Ependyma concept over time: more than just a physical barrier

The ependyma was defined by Purkinje as the epithelium that covers the ventricles and separates the CSF from the brain parenchyma (Purkinje, 1836). The first indications that the ependyma is more than a mere physical barrier appeared more than a 100 years after Purkinje’s description. Brightman and Reese were the first to define the ependyma as responsible for the passage of small molecules and macromolecules between the CSF and the interstitial fluid surrounding the brain parenchyma cells (Brightman and Reese, 1969).

**Figure 5:**
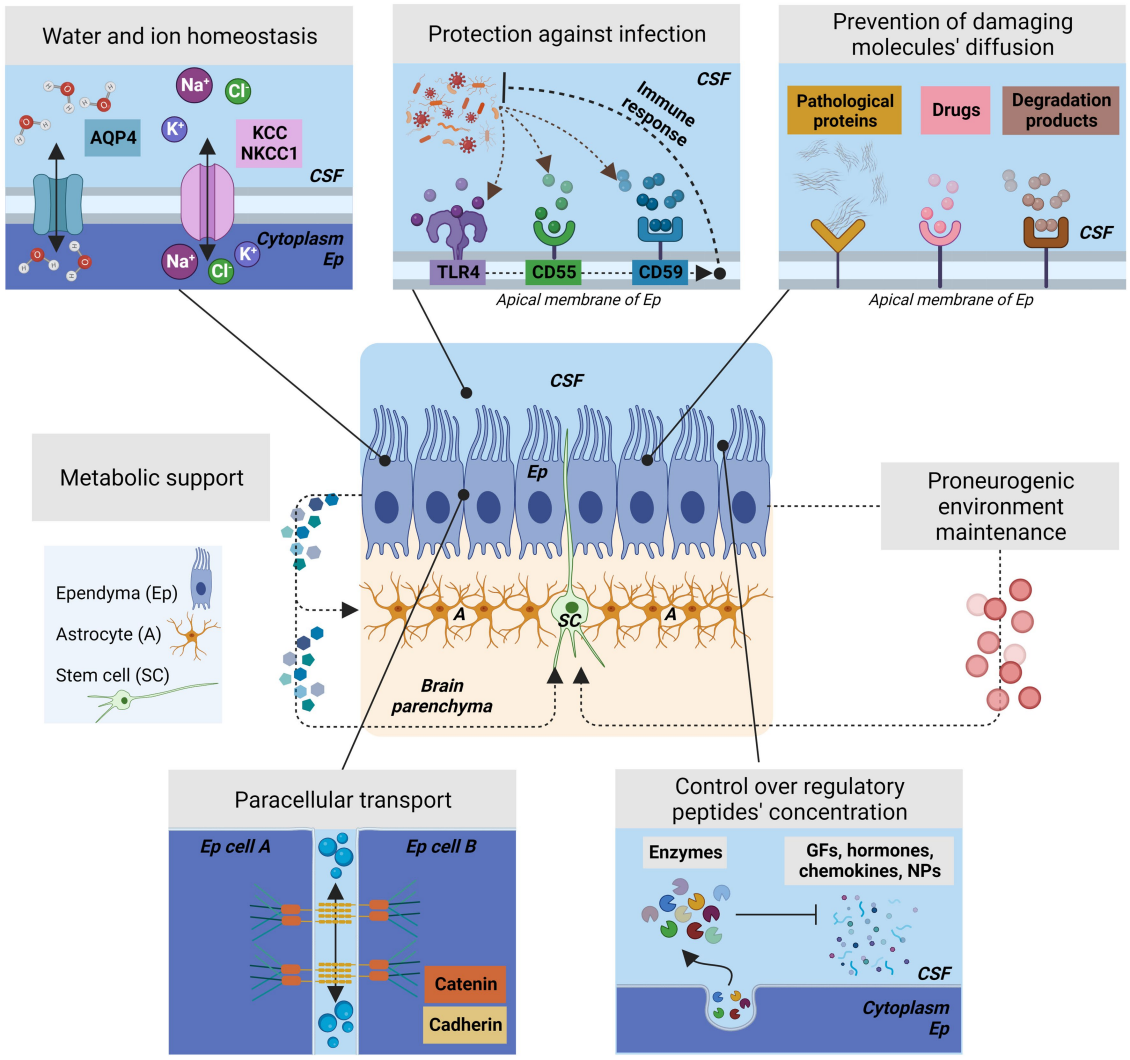
The different roles of ependyma barrier to control the brain homeostasis and neurogenesis. Center, Schematic representation of ependyma epithelium showing the position of the stem cells and the astrocytes located in the subventricular zone. Ependyma provides metabolic support for the astrocytes and stem cells that are in close proximity and will create the proper environment for stem cell function by the various mechanisms represented in this figure. Ep, Ependyma; SC, stem cells; A, astrocytes. Top left, Ependyma actively controls water transport and ion homeostasis by the presence of aquaporins, channels to regulates water flow and osmotic pressure, and different types of cotransporters such as KCC or NKCC1. Top Center, Ependyma plays an active role in preventing infection by expressing receptors in its surface that can activate an immune response against viruses or bacteria. Some representative receptors for this purpose are CD55, or CD59. Additionally, ependyma present components of the complement pathway. The most representative is TLR4. Ependyma will sense and react to infections and will active the nearby microglia. Bottom Left, To control without completely blocking paracellular transport, ependymal cells presents adherent junctions generated by the interactions of cadherins and catenins in a homotypic fashion among apical-lateral surfaces of adjacent ependymal cells; Ependymal cells lack complete tight junctions. These associations are crucial to maintain the differentiation of the CSF and brain parenchyma environments. Bottom Right, Ependyma controls the concentration of surrounding regulatory peptides that are present in the CSF by expressing enzymes that break down the molecules that may affects the ependyma itself or the germinal matrix/subventricular zone. Therefore, ependyma controls growth factors, chemokines, hormones, and neuropeptides on itself and in brain parenchyma. Top Right, Ependyma prevents the diffusion of damaging molecules in the ventricular zone or in brain parenchyma by the presence of receptors located in their membrane. Ependyma presents receptors for drugs, pathological proteins, or enzymatic degradation products. If these products persist, the ependyma function is compromised.

Since then, successive studies have shown that ependyma is not a passive barrier structure but an epithelium that actively regulates the exchange and the flow of ions, signaling factors and metabolites in a controlled manner (Bruni et al., 1985; Jiménez et al., 2014; Saunders et al., 2016; Figure 5). Additionally, ependyma has been implicated in the control of water transport and to the presence of aquaporins (Ma et al., 1994; Venero et al., 1999; Jiménez et al., 2014; Trillo-Contreras et al., 2022), channels to control and regulate water flow (Figure 5). Ependyma also expresses different types of ion cotransporters like K^+^Cl^−^ cotransporter (KCC1) or Na^+^K^+^Cl^−^ cotransporter (NKCC) to provide osmotic control and ion homeostasis (MacAulay and Zeuthen, 2010; Figure 5). Furthermore, to perform this active regulation of the transmembrane transport, lateral cell adhesion is crucial. Ependymal epithelium presents adherent junctions established by cadherins and catenins in a homotypic fashion among apical-lateral surfaces of adjacent ependymal cells (Whish et al., 2015). To allow paracellular transport, ependymal cells lack fully functional tight junctions. The lack of the complete tight junctions allows a high permeability to most small molecules (Jiménez et al., 2014). These two types of junctions are key to the maintenance of the two differentiated environments, the CSF and the neural parenchyma, and provide the proper environment for late brain development and perinatal neurogenesis (Figure 5).

Ependyma has been additionally found to protect the brain parenchyma from damaging circulating molecules, many of them present in inflammatory reactions and related with defects in normal neurogenesis if present during brain development (Figure 5). In this way, ependyma has been described as a structure with the ability to control the concentration of regulatory peptides by expressing enzymes able to break down growth factors, chemokines, hormones, and neuropeptides and, therefore, preventing them from having a deleterious effect in the germinal matrix/subventricular zone and in the brain parenchyma (Gee et al., 1993; Del Bigio, 1995; Figure 5). Similarly, different studies confirmed that ependymal structure prevents the diffusion of harmful molecules from the CSF to the brain. Some specific damaging molecules, such as amyloid-β, drugs, enzymatic degradation products have been found to accumulate at receptors on the ependymal membrane, thus preventing their access to the underlying tissue (Del Bigio, 1995; Fagan and Perrin, 2012; Nelles and Hazrati, 2022; Figure 5). During neuroinflammation, cytokines present in CSF have shown to impair neurogenesis and provoke dysfunction in the neural stem cell niches (Willis et al., 2022; Amanollahi et al., 2023) indicating that the role of ependyma as barrier against damaging molecules is required to maintain normal neurogenesis.

Furthermore, the ependyma has also been described as the first line of defense against infectious agents coming from CSF (Figure 5) as they are expressing receptors on their surface that activate an immune response against viruses or bacteria (e.g., CD55, CD59; Chakravarty and Herkenham, 2005; Canova et al., 2006; Delhaye et al., 2006). Additionally, ependyma expresses components of the complement pathway, Toll-like and non-Toll-Like receptors (Neal et al., 2013). Ependyma seems to also modulate the immune response by controlling the adhesion of intraventricular microglia (Deckert-Schlüter et al., 1994). Microglia are key in the immune process due to the wide range of pattern recognition receptors (PRRs) they display, such as the Toll-like receptors, the NOD-like receptors, receptors for nucleic acids, and C-type lectin receptors (Colonna and Butovsky, 2017). Microglia cluster in the ventricular wall epithelium and in the subventricular zone (VZ/SVZ), and cell death in the ventricular region in the developing forebrain triggers microglial proliferation mediated by the release of macrophage migration inhibitory factor (MIF) that affects to neurogenesis (Arnò et al., 2014). In this same way, if bacterial or viral infection damages the ependyma there is a resulting decline of neurogenesis (Neal et al., 2013; Kamte et al., 2021; Borsini et al., 2022; Stępień et al., 2023), suggesting that the ependyma role in neurogenesis will be affected when ependyma reacts or is affected by infections coming from CSF.

## 5. Ependymal role in neurogenesis

In addition to the homeostatic and regulatory role, ependyma has a major implication in the normal function of the stem cells and, therefore, in the correct course of the neurogenesis during perinatal brain development. The ependyma as epithelium can also provide metabolic support to adjacent parenchymal cells including astrocytes, neural stem cells and different progenitors (Yu et al., 1995; Kobayashi et al., 1996; Silva-Alvarez et al., 2005) and contribute to maintaining the proneurogenic environment of the subventricular zone of the lateral ventricles (Lim et al., 2000; Peretto et al., 2004; Paez-Gonzalez et al., 2011; Figure 5). Disruption of ependyma correlates with reduced neurogenesis in animals and in human cases (Jiménez et al., 2009; Paez-Gonzalez et al., 2011; Rodríguez et al., 2012; Gonzalez-Cano et al., 2016; McAllister et al., 2017; Nascimento et al., 2018). Under pathological conditions, like stroke or hydrocephalus, the immature ependymal epithelium is capable of generating astrocytes and neurons, or even new ependymal cells (Carlén et al., 2009; Bátiz et al., 2011; Redmond et al., 2019). Those findings created confusion about the specific role of multiciliated ependymal cells and the ependyma. For a time, the identity of the neural stem cell was unclear, at times attributed to either the ependymal cells (Johansson et al., 1999) or to the CSF-contacting SVZ astrocytes (Doetsch et al., 1999). After their discovery, the majority of subsequent reports did not support the role of ependymal cells as neural stem cells (Chiasson et al., 1999; Laywell et al., 2000). In culture conditions, ependymal cells were able to proliferate but not to generate neurospheres and neurons; however, SVZ astrocytes were able to proliferate and to generate neurons (Chiasson et al., 1999). Only in pathological conditions, ependymal cells seamed to generate astrocytes and neuroblasts (Carlén et al., 2009). Alvarez-Buylla and Garcia-Verdugo (2002) laboratory, by using *in vivo* retro-viral studies, BrdU administration, [^3^H] Thymidine autoradiography and electron microscopy, were able to demonstrate that the neural stem cells in mature ependyma epithelium were the CSF-contacting SVZ astrocytes (Doetsch et al., 1999; Seri et al., 2001). Other groups confirmed that these neural stem cells shared characteristics with astrocytes (Laywell et al., 2000).

**Figure 6:**
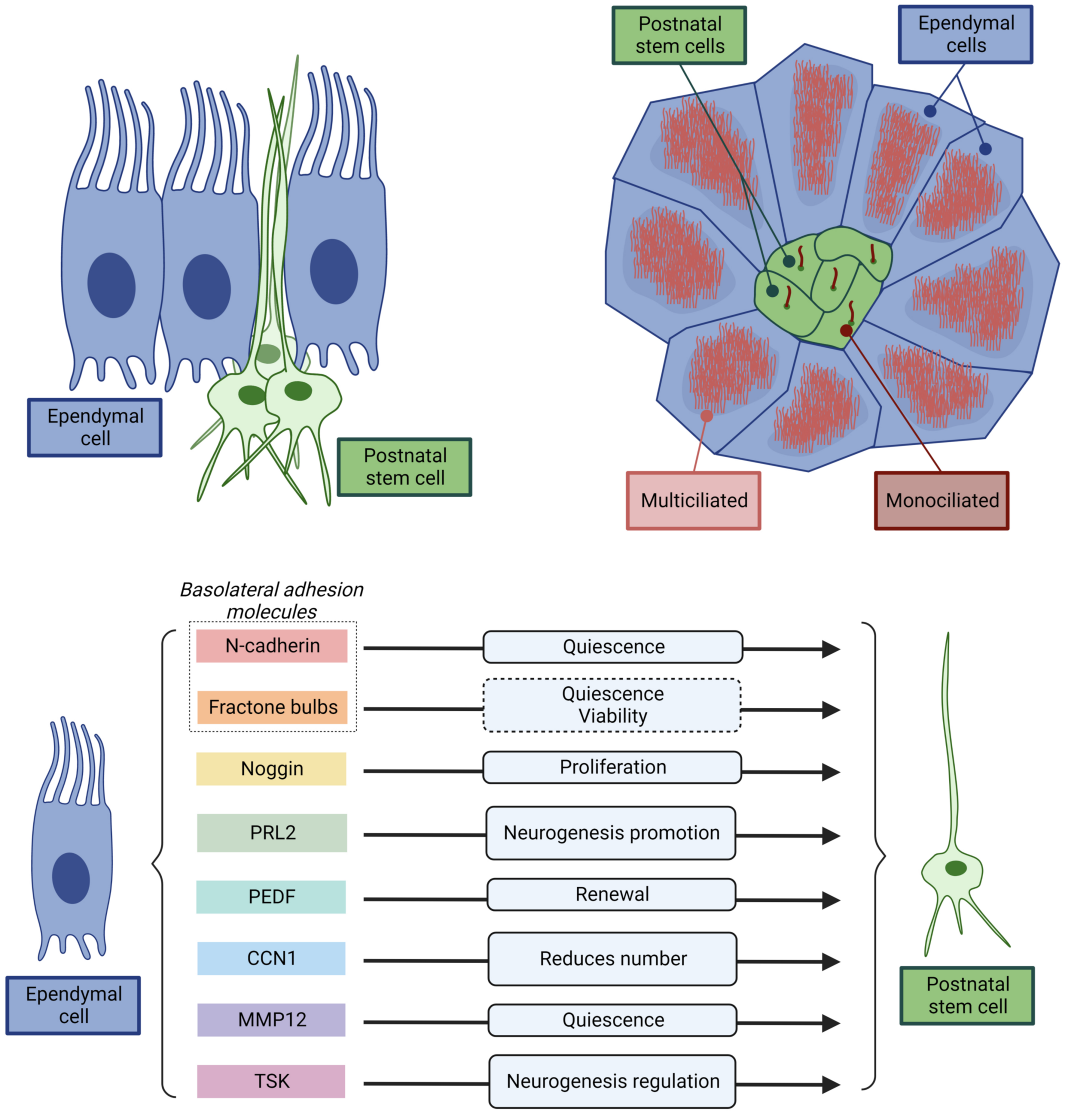
Representative scheme illustrating and summarizing the direct interaction between the multiciliated ependyma and stem cells key for normal neurogenesis. Top left, Cross-section of ependyma epithelium showing the position of the multiciliated ependymal cells and the stem cells. Multiciliated ependymal cells are cuboidal in shape and are distributed throughout the ventricular surface. However, the stem cells have an astrocytic-like shape, and appear in clusters, with the cell body bellow the multiciliated ependymal cells. Only a thin cytoplasmatic extension projects to the ventricular surface. In the apical membrane contacting the CSF, the stem cells present only a single cilium. Top right, Schematic representation of a top-view of ependyma epithelium showing the position of the multiciliated ependymal cells and the stem cells organized in a pinwheel-like structure. The center of each pinwheel-like structure consists of a cluster of stem cells surrounded by multiciliated ependymal cells. This structure ensures a direct and permanent interaction between multiciliated ependymal cells and stem cells that allows to the ependymal cells to exert a decisive control of the stem cell function. Bottom, Summary of the principal mechanisms used by multiciliated ependymal cells to control stem cell function. Both, direct physical interactions through basolateral adhesion molecules (N-Cadherin, and fractone Bulbs), or paracrine signaling (Noggin, PRL2, PEDF, CCN1, MMP12, TSK) and their effects are represented.

In this way, it has been possible to clarify that ependymal cells and neural stem cells located in the brain ventricles are different cell types, even if they share the same origin (radial glial) and location in the ependymal epithelium (Alvarez-Buylla and Garcia-Verdugo, 2002; Redmond et al., 2019). Stem cells are proliferative, morphologically similar to astrocytes, with their cell body located bellow the cell body of the ependymal cells (Seri et al., 2001; Mirzadeh et al., 2008; Kriegstein and Alvarez-Buylla, 2009). Neural stem cells extend a thin extension of their cell body between the multiciliated ependymal cells to reach the CSF (Seri et al., 2001; Mirzadeh et al., 2008; Figure 6). On the other hand, there are several subsets of cells that fit under the terminology of ependymal cells. In addition to the multiciliated ependymal cells, there are also tanycytes (Rodríguez et al., 2005; Yoo and Blackshaw, 2018), and bi-ciliated ependymal cells (Mirzadeh et al., 2008). The multiciliated ependymal cells are postmitotic and do not have a proliferative capacity nor neurogenic activity under normal conditions (Chiasson et al., 1999; Alvarez-Buylla and Garcia-Verdugo, 2002; Spassky et al., 2005; Redmond et al., 2019). Additionally, it has been clarified that the role of multiciliated ependyma is not to produce neurons, but they are necessary to support and control neurogenesis by constituting the neurogenic niche, the microenvironment where the stem cells can properly work at the end of the brain development (Mirzadeh et al., 2008; Paez-Gonzalez et al., 2011; Wu et al., 2020). If the niche is not developed correctly, perinatal and neonatal neurogenesis is lost (Paez-Gonzalez et al., 2011). The niche structure generated by the multiciliated and by bi-ciliated ependymal cells have a specific configuration and organization: the pinwheel-like structure (Figure 6). In the pinwheel-like structure, cell adhesion molecules generate and maintain this structure, and are required for correct performance in neurogenesis (Paez-Gonzalez et al., 2011). For stem cells to mature and differentiate, they must aggregate with their apical cilia through the center of the pinwheels. Quiescent stem cells express vascular cell adhesion molecule 1 (VCAM1) in their apical domain, which is essential for maintaining the structure of pinwheels (Kokovay et al., 2012; Codega et al., 2014; Hu et al., 2017).

In addition to the physical support that ependyma provides to the stem cells, ependymal cells modulate stem cell activity via their cell adhesions: N-cadherin in the basolateral membrane between ependymal cells and neural stem cells promotes quiescence of the latter (Porlan et al., 2014; Figure 6). The ependyma regulates the adhesion of neural stem cells by producing round structures called fractone bulbs, which are part of the extracellular matrix to which neural stem cells adhere and could promote cell quiescence and viability (Nascimento et al., 2018). Therefore, basolateral ependymal cells-stem cell adhesion molecules allow the ependyma promote cell quiescence and viability of stem cells.

Ependymal cells also regulate the activity of neural stem cells through paracrine signals (Figure 6). They secrete Noggin, which stimulates the activation and proliferation of neural stem cells, while antagonizing the effect of bone morphogenetic protein (BMP), which inhibits neurogenesis (Lim et al., 2000; Yousef et al., 2015). Another molecule secreted by ependymal cells that antagonizes the effect of BMP is low-density lipoprotein receptor-related protein 2 (PRL2), whose effect is to promote neurogenesis (Gajera et al., 2010). Other molecules secreted by ependymal cells are: the pigment epithelium-derived factor (PEDF), which promotes the renewal of neural stem cells (Ramírez-Castillejo et al., 2006); cell communication network factor 1 (better known: CCN1), that reduces the number of stem cells (Wu et al., 2020); matrix metalloproteinase-12 (MMP12), which promotes stem cell quiescence (Shan et al., 2018); and Tsukushi (TSK), a leucine-rich proteoglycan that regulates several signaling pathways related to neurogenesis, binding to receptors or ligands of these pathways (Ohta et al., 2004, 2011; Kuriyama et al., 2006). Additionally, the cilia that ependymal cells present in their apical surface help to create the appropriated gradients of molecules in the CSF that direct the migration of neuroblasts (Sawamoto et al., 2006).

Therefore, even if multiciliated ependymal cells are not stem cells, ependyma as epithelium has a direct role in neurogenesis reflected in the pinwheel-like structure, the metabolic support to adjacent cells, the regulatory role by direct cell–cell interaction, and the regulatory role by secreting molecules to target stem cell activity. Pathological disruption of this structure during development after GMH/IVH will be manifested in defects in neurogenesis whose consequences are long-term neurological deficits (Tsitouras and Sgouros, 2011; Pindrik and Halverson, 2019).

## 6. Neuroinflammatory response to GMH/IVH

**Figure 7:**
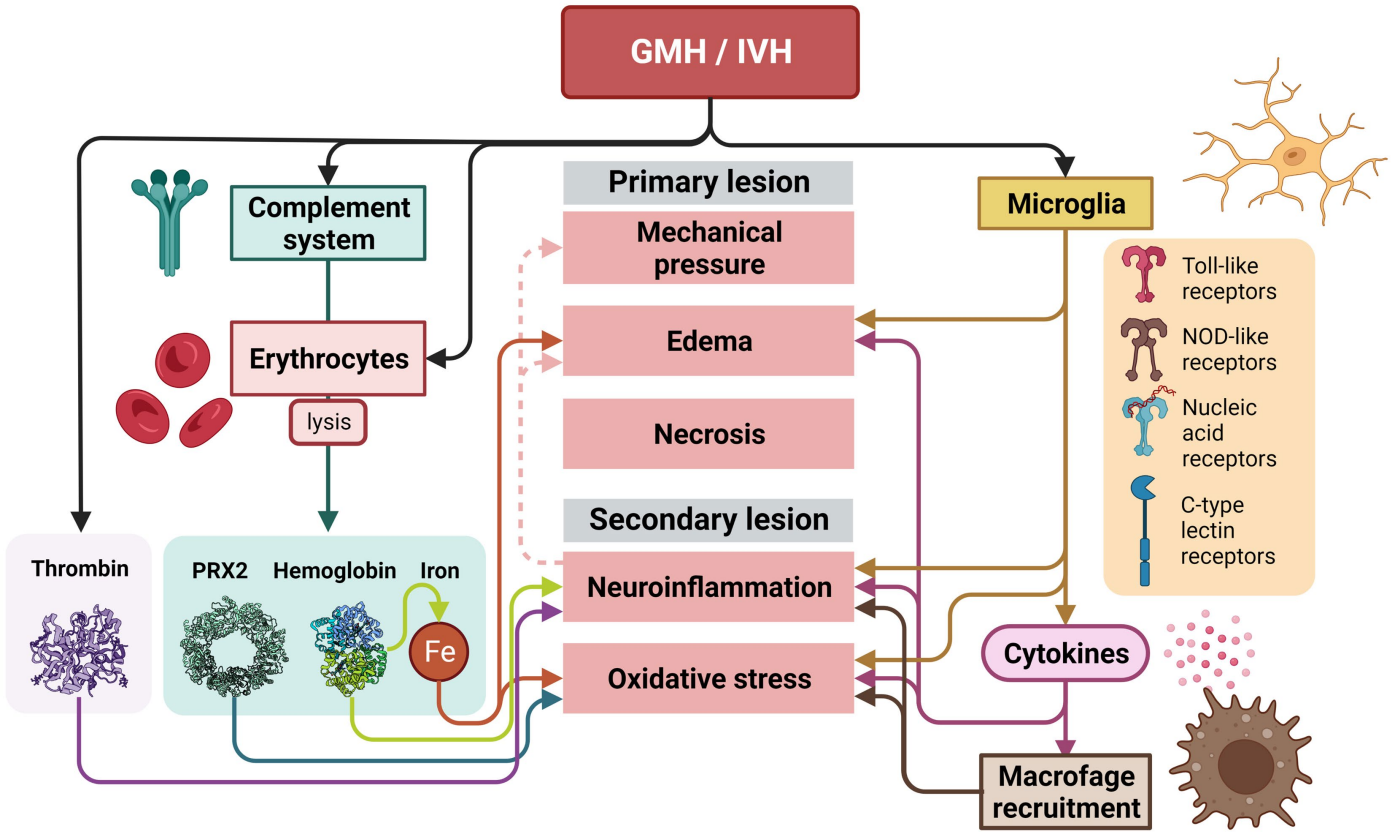
Inflammatory response that takes place after a GMH/IVH in the germinal matrix and ependyma. After the GMH/IVH, erythrocytes are released into the ventricular system. Lysed erythrocytes will release their potentially neurotoxic components (PRX2, hemoglobin, and iron), that together with thrombin released into the CSF after the hemorrhage will induce and the potentiate primary and secondary lesion. The primary and secondary lesions are also initiated and potentiated by the microglia activation. Activation of microglia occurs after the GMH/IVH by the Toll-like receptors, the NOD-like receptors, receptors for nucleic acids, and C-type lectin receptors. The release of cytokines will strongly increase neuroinflammation, edema and oxidative stress. Neuroinflammation will also increase edema and mechanical pressure over time.

Perinatal intraventricular hemorrhages correlate with ependymal damage (McAllister et al., 2017). Studies show how the severity of the inflammatory response associated to GMH/IVH also correlates with the severity of the ependymal damage (Dawes, 2022). Following GMH/IVH, erythrocytes are released into the ventricular system, they lyse, and their potentially neurotoxic components are released into the CSF (Klebe et al., 2015; Tsuji et al., 2020). The hemorrhage applies mechanical pressure on glia and neurons, giving rise to cytotoxic edema and necrosis, which is known as primary lesion, followed by neuroinflammation and oxidative stress, known as secondary lesion (Klebe et al., 2015; Tsuji et al., 2020; Figure 7).

The primary lesion activates microglia as primary response to ischemia. Once activated, microglia phagocytose red blood cells and secrete proinflammatory cytokines, extracellular proteases, and oxidative species (Klebe et al., 2015; Tsuji et al., 2020). Microglia are key in this process due to the wide range of pattern recognition receptors they display, such as the Toll-like receptors, the NOD-like receptors, receptors for nucleic acids, and C-type lectin receptors (Colonna and Butovsky, 2017). Cytokines released by the microglia have a wide effect: increasing vascular permeability, promoting the formation of cerebral edema, recruiting leukocytes, and have neurotoxic effects (Ziai et al., 2021). Additionally, cytokines produce oxidative stress (Bejar et al., 1983; Leviton et al., 1999; Sävman et al., 2002), thus neural synapse formation is damaged, myelination becomes defective, and neurotransmitters are altered (Papile et al., 1978; Del Bigio, 1993; Roland and Hill, 1997; Ziai et al., 2021; Figure 7).

Finally, the complement system, an innate immune response constituted by an assortment of serum proteins is involved. The complement system senses and respond to certain stimuli such as antibodies, bacterial carbohydrates, apoptotic or hypoxic cells, etc. (Pouw and Ricklin, 2021). The complement system is responsible for causing lysis of erythrocytes after GMH/IVH and attracts macrophages to the damaged area (Mevorach et al., 1998; Wang et al., 2019; Tan et al., 2020; Chen et al., 2021). Some of the components that are released into the CSF when erythrocytes lyse during this second phase are hemoglobin (Hb), iron, and peroxiredoxin-2 (Huang et al., 2002; Bian et al., 2020; Chen et al., 2021) (Prx2). Prx2 is a potent proinflammatory factor causing macrophage activation, ependymal damage, and hydrocephalus (Tan et al., 2020; Chen et al., 2021). These components together with plasma proteins, including thrombin, can cause more inflammation, leading to further brain damage (Gao et al., 2014; Wilkinson et al., 2018; Peng et al., 2021). Hemoglobin is a potent activator of inflammation. Hemoglobin dissociates and enters cells by endocytosis, where its heme group is degraded and iron is released, which can cause oxidative damage to nearby tissue (Iwanowski and Olszewski, 1960; Nakamura et al., 2005; Lee et al., 2010; Liu et al., 2017). Iron accumulation is associated with ventricular dilation and cerebral edema (Dai et al., 2019). Iron is also involved in ventricular dilatation and fibrosis and CSF hypersecretion (Meng et al., 2015), and together with hemoglobin induce ventricular distent (Strahle et al., 2014). Additionally, thrombin, a protease that increases its levels after GMH/IVH induces blood coagulation and activates inflammation through ischemia (Chen and Dorling, 2009; Figure 7).

Due to the proximity of the ependyma to the germinal matrix (Figure 1), blood and blood degradation products after hemorrhage in the germinal matrix will diffuse into the ependyma. Therefore, primary and secondary lesions will also reach the ependyma and all the inflammatory molecules will be in contact with the multiciliated ependymal cells (Figures 1, 3).

## 7. Maintaining mature ependyma

Different studies have shown that ependymal cells change and de-differentiate and/or proliferate under pathological conditions, such as those triggered by GMH/IVH or bacterial/viral infection (Carlén et al., 2009; Bátiz et al., 2011). However, the molecular mechanism behind this change in the nature of multiciliated cells has not been well explained. Surprisingly, recent studies show that, unlike other differentiated cells, the mature status of ependymal cell needs to be continuously and actively maintained, and that interaction with inflammatory molecules will lead to the loss of that mature state (Abdi et al., 2018). In this process, the Foxj1 transcription factor has a key role. Foxj1 is a known regulator of cilia formation and is expressed in radial glial cells committed to ependymal fate (Brody et al., 2000; Jacquet et al., 2009). Interestingly, Foxj1 transcription factor has a short half-life and is degraded by the ubiquitin proteosome system. If Foxj1 is not maintained, ependymal cells dedifferentiate into cells with a morphology similar to radial glial cells. Therefore, this factor needs to be constantly produced by ependymal cells to remain differentiated (Abdi et al., 2018). In addition, to avoid its rapid degradation, Foxj1 requires phosphorylation by IKK2 (the kinase complex subunit 2) in an IKKγ/NF-KB-independent manner to prevent its degradation by ubiquitin proteosome system (Abdi et al., 2018).

**Figure 8:**
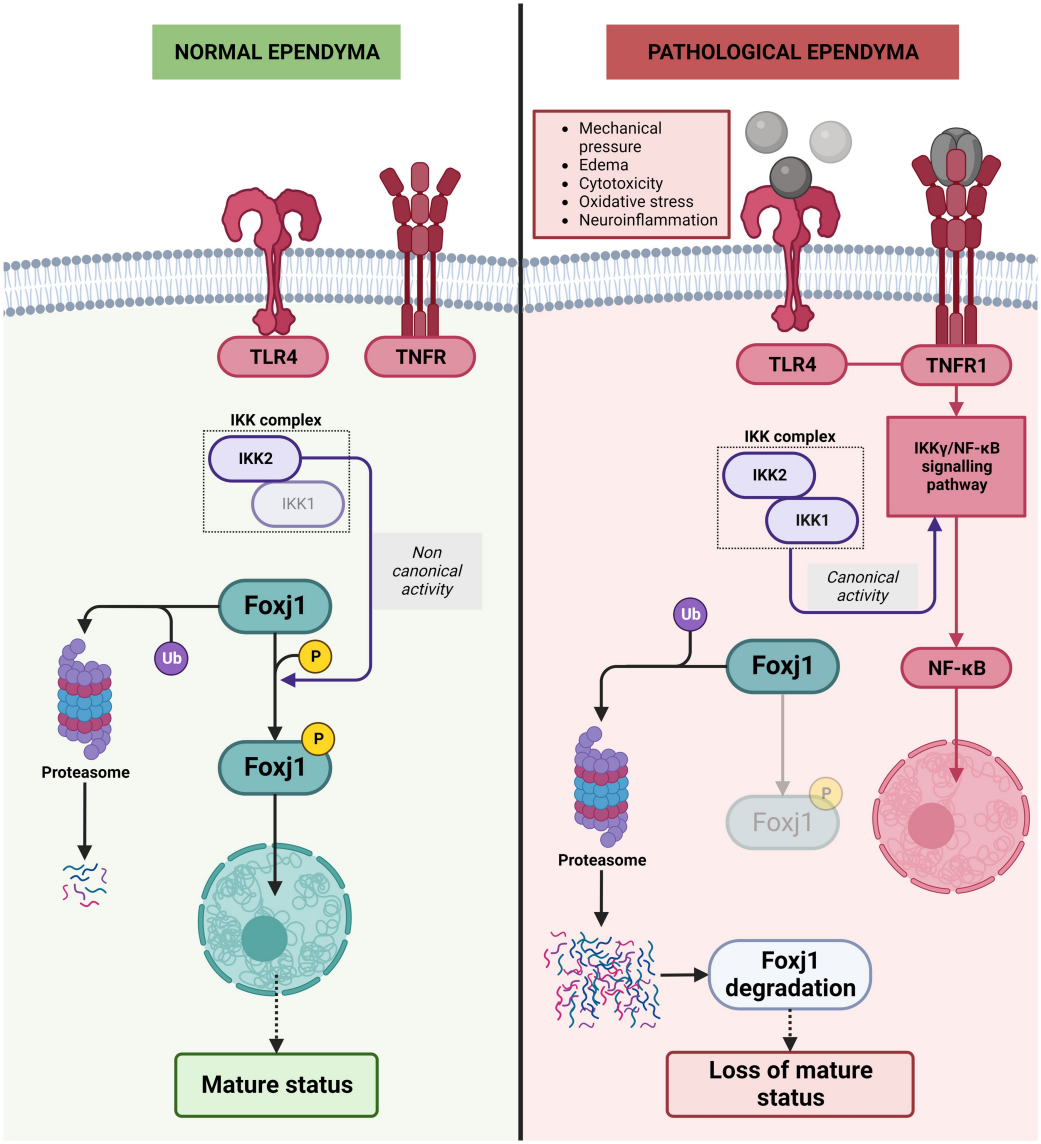
Molecular mechanisms behind the de-differentiation of ependymal cells during neuroinflammation. In normal ependyma, phosphorylation of Foxj1 transcription factor by IKK2 complex (the kinase complex subunit 2) in a non-canonical manner (IKKγ/NF-KB-independent manner) prevents its degradation by ubiquitin proteosome system and allows the Foxj1 transcription factor to reach the nucleus to maintain mature ependyma status. Under pathological conditions/inflammatory conditions, the TLR4 pathway is activated, IKKγ/NF-KB canonical activity triggered and phosphorylation of Foxj1 transcription factor is reduced, Foxj1 transcription factor is degradated by the ubiquitin proteosome system and loss of mature status occurs.

The IKK2 is part of the IKK complex that contains 2 protein kinases, IKK1 (IKKα) and IKK2 (IKKβ). After an inflammatory stimulus i.e., blood degradation products, cytokines, oxidative stress, cytokines, and neurotoxins, IKK2 is also the critical kinase subunit inducing the canonical signaling pathway (IKKγ/ NF-κB pathway) involved in the regulation of inflammation and cell survival (Mémet, 2006; Perkins, 2007; Oeckinghaus et al., 2011; Hayden and Ghosh, 2012). In the presence of inflammatory stimuli, IKK2 is no longer stabilizing Foxj1 transcription factor, and is mostly degraded by the ubiquitin proteosome system (Abdi et al., 2018; Figure 8). One of the major players in this process is TLR4. Blood degradation products have the ability to bind TLR4 and induce NF-κB to be translocated to the nucleus and initiate production of proinflammatory cytokines such as TNF-α or IL-6, chemokines, as well as trigger reactive oxygen and nitrogen species (Bryant et al., 2010; Zusso et al., 2019). In ependymal cells, TLR4 are also present (Chakravarty and Herkenham, 2005) and when signaling pathway is activated, ankyrin-3 (Ank3), a critical adapter protein found in mature ependyma, is decreased (König et al., 2017; Figure 8).

TLR4 is also highly expressed in microglia, which responds to blood degradation products, and is characteristic of the damage-associated molecular patterns (DAMPs), such as lysophosfatidic acid (LPA), iron, and methemoglobin (Karimy et al., 2020). Production of proinflammatory cytokines such as TNF-α or IL-6, chemokines, and reactive oxygen and nitrogen species are triggered and enhanced (Bryant et al., 2010; Zusso et al., 2019). TNF-α has a pro-inflammatory role when bound to its receptor, TNF-α receptor1 (TNFR1) (Probert, 2015; Figure 8). Additionally, TNFR1 has been recently identified in the ependymal cells in *in-vitro* experiments (Fernández-Arjona et al., 2021). When TNFR1 is stimulated, the NF-κB pathway is activated (Hayden and Ghosh, 2014) inducing the degradation of Foxj1 transcription factor, loss of cilia and therefore results in ependymal cell de-differentiation (Abdi et al., 2018).

## 8. Short-time consequences of de-differentiation caused by activation of inflammatory pathways

**Figure 9:**
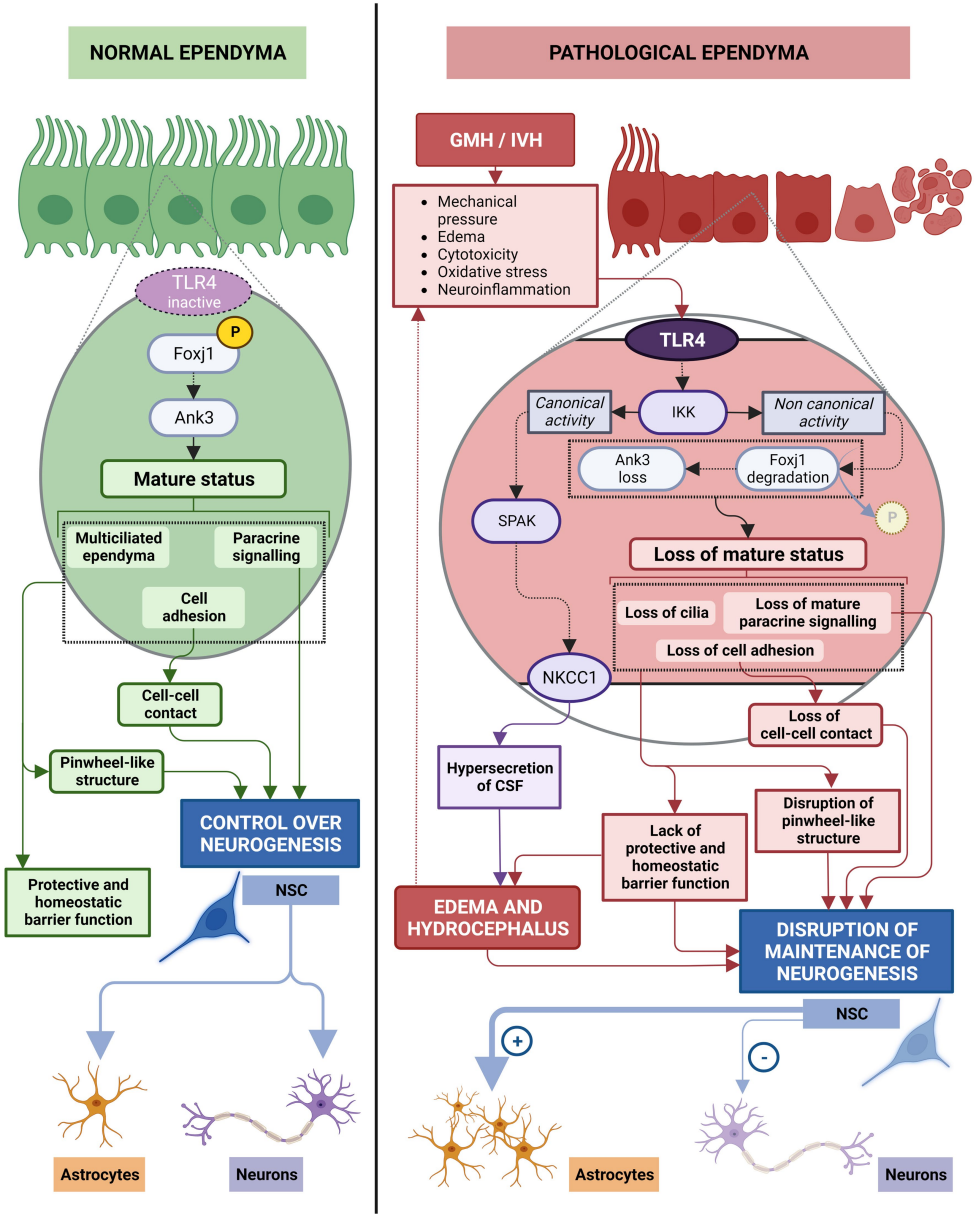
The role of ependyma on neurogenesis in normal and neuroinflammation induced by GMH/IVH conditions. In healthy ependyma, phosphorylation of Foxj1 transcription factor through non-canonical IKK2 signaling (IKKγ/NF-KB-independent manner) activates Ank3 pathway to maintain the mature multiciliated ependyma. In this status, ependymal cells exert direct control on neurogenesis by maintaining the pinwheel-like structure, by cell–cell contacts through basolateral adhesion molecules (N-Cadherin, and fractone Bulbs), and by paracrine signaling (Noggin, PRL2, PEDF, CCN1, MMP12, TSK). Additionally, ependymal cells maintain the appropriated microenvironment for stem cell function through its protective and homeostatic barrier function. In this conditions, neural stem cells produce the needed progeny. After GMH/IVH, TLR4 pathway is activated, IKKγ/NF-KB canonical activity triggered, Foxj1 transcription factor is reduced and its degradation by ubiquitin proteosome system is potentiated, initiating loss of mature status. Pinwheel-like structure disappears, control of stem cell function by cell–cell contacts through basolateral adhesion molecules is altered and the paracrine control of stem cell function becomes defective. The microenvironment that ependymal cells generate for proper stem cell function is disrupted and homeostatic barrier function of ependyma is lost. Additionally, IKKγ/NF-KB canonical activity induces SPAK activation that leads to NKCC1 upregulation and hypersecretion of CSF, contributing to edema and hydrocephalus, that will further alter the microenvironment. Neural stem cells produce mostly astrocytes under these pathological conditions.

Foxj1 degradation induced by activation of inflammatory pathways will lead to alteration of different adhesion molecules. Ank3 protein in particular, has been described as a main player in organizing membrane domains and is required for the formation of the pinwheel-like structure development in ependyma. Ank3 protein is located at the lateral borders of ependymal cells (Bennett and Healy, 2008) but it is not present in neural stem cells (Paez-Gonzalez et al., 2011; Figure 9).

The de-differentiation process caused by Foxj1 degradation after activation of inflammatory pathways results in reduction of Ank3 expression, loss of cilia, modification of cell adhesion molecules and cell junctions, and loss of differentiated status (Paez-Gonzalez et al., 2011; Abdi et al., 2018; Figure 9). Mature cell adhesion molecules have been described to be one of the mechanisms used by mature ependymal cells for exerting control over the stem cells (Porlan et al., 2014; Nascimento et al., 2018) as well as paracrine signals (Lim et al., 2000; Ohta et al., 2004; Kuriyama et al., 2006; Gajera et al., 2010; Ohta et al., 2011; Yousef et al., 2015; Wu et al., 2020; Figure 6).

Therefore, short-term consequences of the de-differentiation of ependymal cells include loss of ependymal regulation of stem cell function. In this way, it has been described that, when Foxj1 and Ank3 levels are reduced in the ependymal cells, stem cells stop differentiating into neurons and start to differentiate into astrocytes (Paez-Gonzalez et al., 2011; Figure 9). In healthy conditions, neuron production should be maintained at least 6 months after birth in the lateral ventricles in humans (Sanai et al., 2011). Consequently, an important short-term consequence is a deficit in normal neonatal neurogenesis that may have important consequences for normal brain and cognitive development (Figure 9).

In addition to implications on neurogenesis, de-differentiation of ependyma can exacerbate CSF overproduction leading to PHH. The complex inflammatory and de-differentiation signaling pathway involving canonical TLR4-IKK signaling (Kaltschmidt et al., 2005; Mattson, 2005; Hayden and Ghosh, 2012; Ledoux and Perkins, 2014) is mediated by transcription of the proline/alanine-rich kinase (Yan and Merlin, 2008) (SPAK). SPAK phosphorylates sodium-potassium-chloride cotransporters (NKCC1) that then translocate to the apical membrane of ependymal cells (Thastrup et al., 2012; Alessi et al., 2014; Karimy et al., 2017). NKCC1 activation causes an upregulation of aquaporin 4 (AQP4) expression. AQP4, located on the basolateral membrane of ependymal cells (Nielsen et al., 1997; Rash et al., 1998), has an important role in the removal of excess CSF (Bloch et al., 2006; Figure 9). This aquaporin is the most abundant in the brain and can be present as a short isoform (called M23) or as a long isoform (Neely et al., 1999) called M1. In hydrocephalus, the main function of AQP4 is compensatory: functioning as an alternative pathway for CSF uptake through ependymal cells (Mao et al., 2006; Xi et al., 2006). The NKCC1-AQP4 cascade is dependent on NF-κB and SPAK, such that if NF-κB is altered, AQP4 overexpression occurs (Gram et al., 2013, 2014). If this is maintained over time, it will cause CSF hypersecretion (Gram et al., 2013, 2014) and AQP4 overexpression (Gram et al., 2013, 2014) that contributes to parenchymal edema and hydrocephalus (Figure 9). This leads over time to a dangerous increase in intracranial pressure (ICP), disruption to neuronal tissue and, therefore, to PHH and a long-term brain injury (Chen et al., 2017).

## 9. Risks of untreated ependymal damage

The role of the ependyma is critical for the correct flow and circulation of the CSF (Brightman and Reese, 1969; Jiménez et al., 2014). Sustained damage to the ependyma over time has drastic consequences for maintenance of normal neurogenesis and long-term homeostasis of the CNS. The damaged ependyma becomes the center of a cascade of events that causes hydrocephalus and neurogenesis defects that will continue in an amplifying loop if not treated (Figure 9).

**Figure 10:**
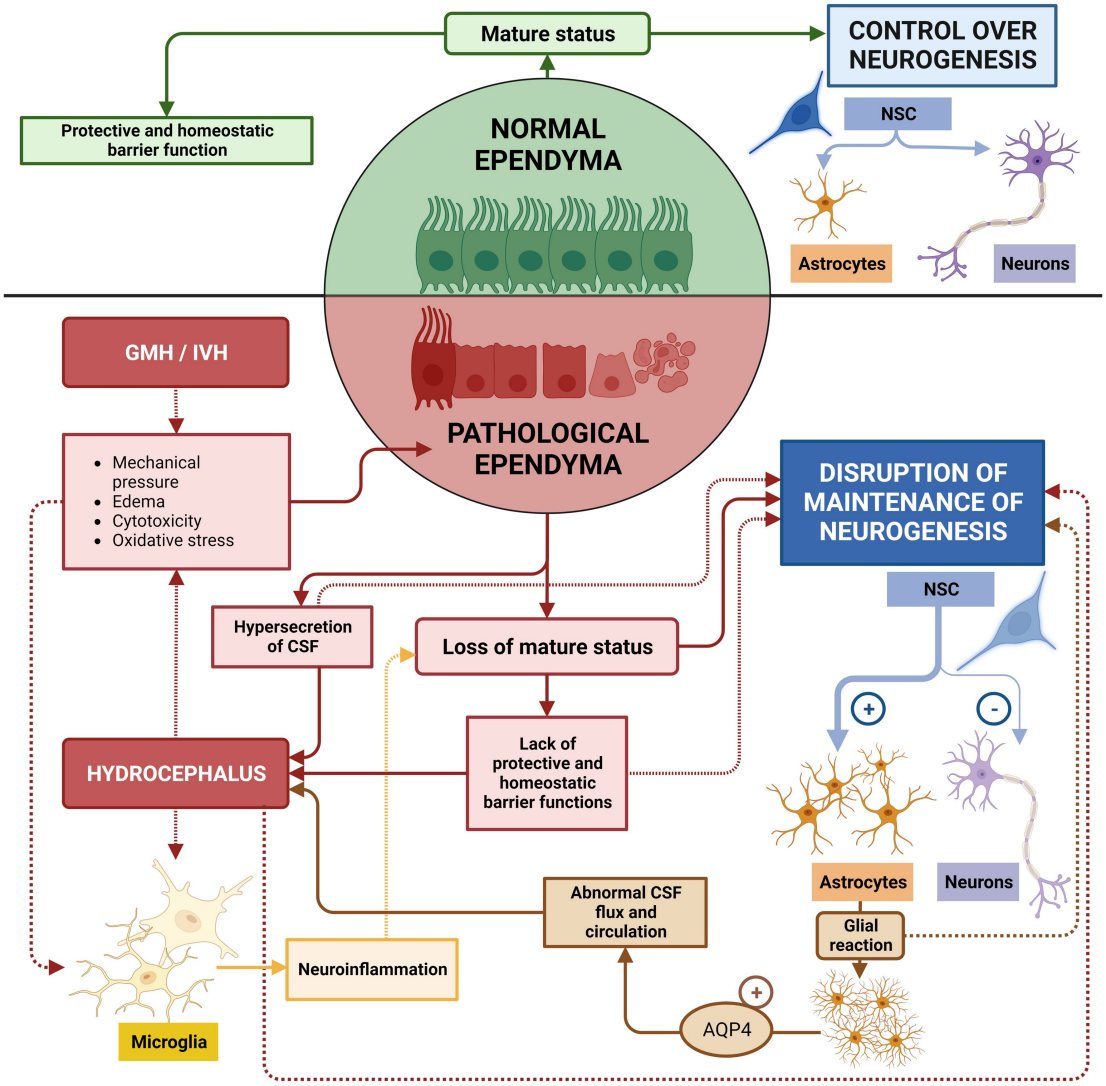
Risks of untreated ependymal damage. In healthy conditions, normal ependyma maintains a mature status capable of regulating brain homeostasis and sustaining neurogenesis. If GMH/IVH occurs, neuroinflammatory conditions induce the de-differentiation of mature ependyma, neurogenesis defects and edema/hydrocephalus. Additionally, mature ependyma discontinuities in the ependymal lining, which then induces short and mid-term proliferation of periventricular astrocytes, preventing proper function of remaining ependyma and, therefore, to the normal stem cell function. Astrocytes additionally increase expression of AQP4 that contributes to abnormal CSF flux and circulation, which aggravates edema and hydrocephalus. These two phenomena will increase cytotoxicity, mechanical pressure, and oxidative stresses that will potentiate inflammatory reactions, and will increase ependyma damage and periventricular astrocytic reaction.

Ependyma de-structuration induces short and mid-term proliferation of periventricular astrocytes in a periventricular astroglia reaction (Vizuete et al., 1999; Mao et al., 2006; Roales-Buján et al., 2012; Figure 10). A population of astrocytes, reactive astrocytes, form a new layer that acts as a barrier between the CSF and the brain parenchyma, replacing the lost ependyma (Roales-Buján et al., 2012; McAllister et al., 2017; Castaneyra-Ruiz et al., 2018) and will stay for long-term. This new layer shares phenotypic and functional characteristics with the ependyma: numerous microvilli project into the ventricles, high expression of aquaporin 4, endocytosis mechanisms, and a similar paracellular pathway for CSF absorption (Roales-Buján et al., 2012). However, they do not perform the functions of circulation and filtering with the same efficiency, which results in an abnormal flow of the CSF. This is associated with abnormal brain development, enlarged ventricles, and hydrocephalic edema (McAllister et al., 2017; Castaneyra-Ruiz et al., 2018; Figure 10). The new layer of reactive astrocytes presents a high expression of AQP4, which seems to have the same function as in the ependyma (Vizuete et al., 1999; Mao et al., 2006; Xi et al., 2006; Roales-Buján et al., 2012). Finally, due to the persistent inflammation that causes the astrocytes to react, new layers of astrocytes continue to form around the remaining ependyma and prevent their proper functioning (Roales-Buján et al., 2012; García-Bonilla et al., 2023; Figure 10).

Additionally, the de-structuration of the ependyma epithelium, alter the functions of ependyma as barrier epithelium. In this way, the control of diffusion of ions, signaling factors, metabolites (Bruni et al., 1985; Jiménez et al., 2014; Saunders et al., 2016), the breakdown of growth factors, chemokines, hormones, and neuropeptides to prevent a deleterious effect on the brain parenchyma (Del Bigio, 1995) the blocking of harmful molecules from the CSF(Del Bigio, 1995; Fagan and Perrin, 2012), and the immunological role of the epithelium preventing the progression of infections (Chakravarty and Herkenham, 2005; Canova et al., 2006; Delhaye et al., 2006), disappear or is disrupted (Figure 10).

Therefore, de-differentiation of the ependyma will have short–and mid-term consequences on the stability of the epithelium, on neurogenesis, and on the developing ventricular wall. Any therapy targeted to reduce inflammatory response in the bleeding area after the GMH/IVH may have a great short and mid-term impact in the stability of the brain parenchyma, and will have an important clinical impact, but will need to pay attention to the long-term disruption of the ependyma in order to minimize the long-lasting neurological deficits in cognitive and psychomotor abilities (McCrea and Ment, 2008; Tsitouras and Sgouros, 2011; Pindrik and Halverson, 2019).

## 10. From conventional therapies to the use of stem cells

**Figure 11:**
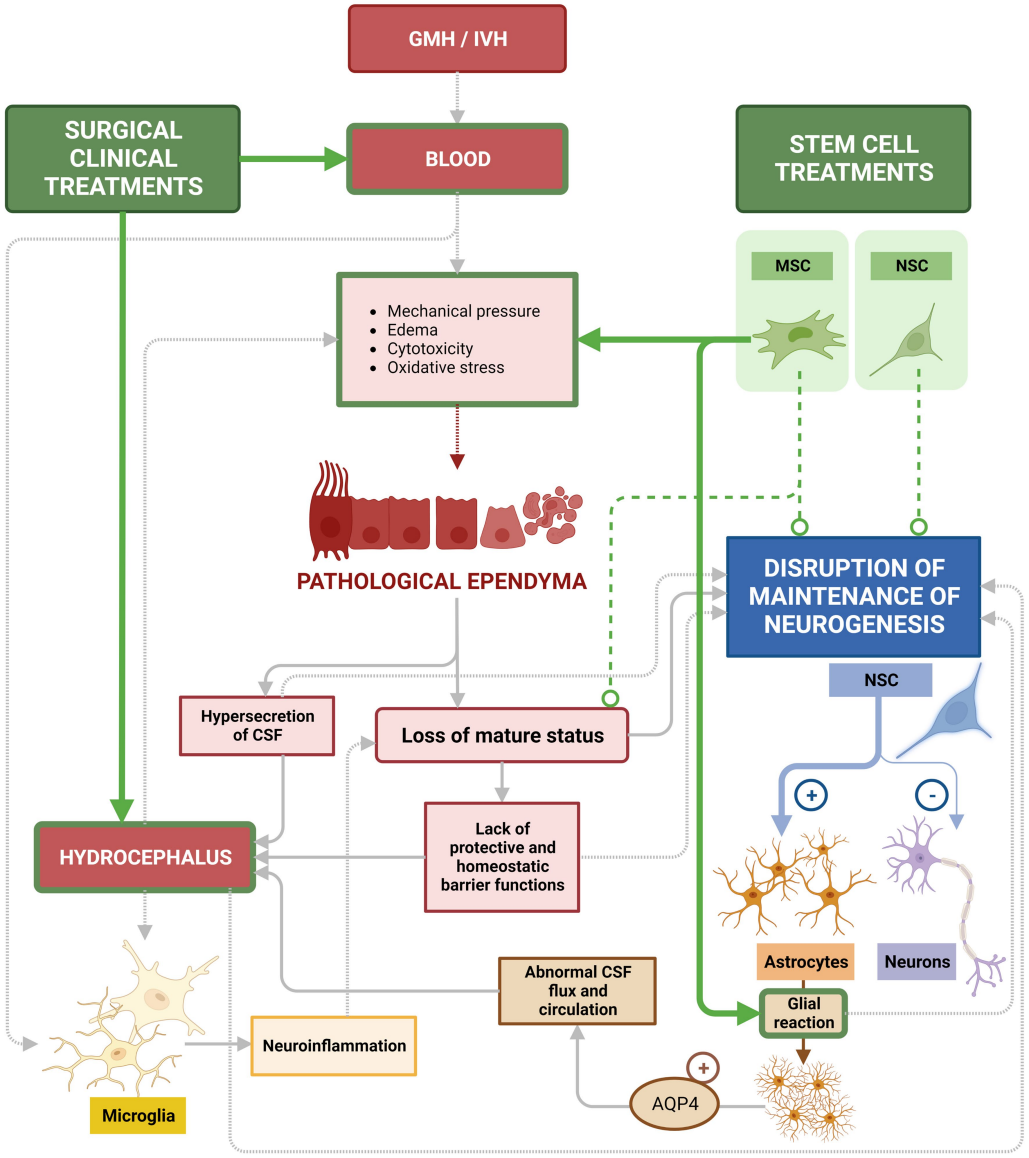
Current treatments used to treat GMH/IVH and PHH. Surgical interventions to treat GMH/IVM/ and PHH aim to alleviate ventricular pressure by drainage of CSF and blood to reduce the risk of hydrocephalus. Those treatments (green arrows) are efficient for their specific purpose, but neglect to treat or recover the underlying ependymal damage that persists. In the same way, these treatments are not directed to treat or recover the lost neurogenesis aspect, critical for proper neonatal development. Stem cell-based treatments with MSCs are efficient in reducing inflammatory conditions after bleeding (green arrows), including a marked reduction of astroglia reactions (green arrow). However, attempts using MSCs to recover the ependyma or restore neurogenesis have not yet been successful (dashed line). While introduced NSCs do integrate into the ventricular wall, no efficient treatment based on NSCs has been successful in restoring neurogenesis (dashed line). Therefore, loss of mature ependyma status and the consequent alteration in neurogenesis is still lacking treatment and is the main gap remaining to be addressed.

Current treatments used to treat GMH/IVH and PHH are directed to remove the blood and alleviate ventricular pressure by drainage of CSF through intraventricular catheters, external drains, endoscopic ventricular lavage, or lumbar punctures (Romero et al., 2014). In lumbar puncture, excess CSF is removed using a needle to directly drain the excess of CSF to avoid its accumulation, through a reservoir, until the infant reaches an age sufficient to accept an efficient derivation without risks (Whitelaw and Lee-Kelland, 2017; Joyce et al., 2019). Other treatments aim to reduce the motor and cognitive damage through a DRIFT protocol (drainage, irrigation, and fibrinolytic therapy). In this case, endoscopic ventricular lavages can be a useful tool in reducing the ventricular blood load and its degradation products (Joyce et al., 2019). Endoscopic ventricular lavage removes blood and blood breakdown products (such as iron or Prx2) accumulated in the ventricles due to GMH/IVH (Song et al., 2018). Diverting the CSF may be temporary or permanent, depending on the infant’s ability to tolerate treatment (Ellenbogen et al., 2016). These temporary measures may reduce the need for permanent CSF diversion in a subset of infants with PHH, but many of these preterm infants will require definitive measures to treat hydrocephalus (Lam and Heilman, 2009; Wellons et al., 2009; Wang et al., 2014; Figure 11).

Unfortunately, current treatments present some inconveniences: CSF leakage during the process, infection, or device malfunction are common (Wang et al., 2014; Ellenbogen et al., 2016; Kulkarni et al., 2016). Additionally, the primary deficiency, the lack of attention to the ependymal recovery, remains (Figure 11). Therefore, a major gap in current treatments is the lack of strategies to recover damaged ependyma and normal neurogenesis in long term (Figure 11).

New medical technologies in cellular therapies could change the way that GMH/IVH and hydrocephalus are treated and can complement the current CSF diversion surgeries. In recent years, the interest in utilizing stem cells to design new therapeutic options for treating neurodegenerative diseases has increased due to the regenerative ability of these cells (McKay, 2000; Ahn et al., 2013).

Cell-based therapies have reasonable therapeutic merit due to the regenerative potential inherent in stem cells. Stem cells by themselves are not sufficient to recover after a GMH/IVH. However, they can be used together with the conventional surgical therapies based on draining and diverting the CSF. Preclinical and clinical trials using stem cell-based treatments for GMH/IVH are relatively recent, therefore current applications remain technically incomplete or incite ethical controversy (Ahn et al., 2013).

## 11. Treating GMH/IVH and PHH with new therapeutic strategies

Different subsets and sources of stem cells have been attempted to be used as tools to treat GMH/IVH and PHH. Embryonic stem cells have been used to treat congenital hydrocephalus has given promising results (Guerra, 2014). Attending to their potential to give rise to different cell types, embryonic stem cells present the highest potential. However, their use entails ethical problems since they need to be obtained from embryos (Guerra et al., 2017). Additionally, appearance of tumors after transplantation remains a considerable risk (Sandel, 2004).

Over the last decades, other multipotent and pluripotent stem cells have been proven to be more reliable tools with less ethical inconveniences. Among the most promising of these is the research with induced pluripotent stem cells (iPSC). iPSC are generated from adult somatic cells and their pluripotency is artificially induced through the sequential expression of different transcription factors (Takahashi et al., 2007). iPSC are also frequently used in laboratory settings to reproduce and study disease models and for medical transplantation (Kim et al., 2010). iPSC similarly appear promising but have not been tested for GMH/IVH/PHH.

Mesenchymal stem cells (MSC) derived from bone marrow or umbilical cord have been found to have a significant therapeutical potential due to their immunomodulatory and neuroprotective effects after transplantation (Mayani, 2003; Chang et al., 2007; Parr et al., 2007). Those characteristics make them ideal for the treatment of CNS diseases mediated by the immune system, such as neurodegenerative diseases (Sadan et al., 2009), GMH/IVH or hydrocephalus (García-Bonilla et al., 2020). MSC generate favorable environments for regeneration, promote vascularization and myelination (Parr et al., 2007; Lee, 2012; Han et al., 2016).

Clinical trials for treating GMH/IVH/PHH with stem cell are recent and are mainly focused on the use of MSC as tool to reduce inflammatory conditions. These studies thus far support the safety of the use of MSC and their efficacy in alleviating neurological deficits in patients with GMH/IVH (Sadan et al., 2009; Ahn et al., 2013; Park et al., 2016; García-Bonilla et al., 2020). In particular, MSC did not induce mortality among the patients and patients showed attenuation in the periventricular hemorrhagic infarction and a regression in the hemorrhage (Ahn et al., 2013). In animal models of GMH/IVH/PHH and hydrocephalus, MSC cells have been more deeply studied. MSC from human umbilical cord blood have been able to prevent PHH, protect against HIV-induced brain damage, and decrease the concentrations of inflammatory cytokines such as TNF-α or IL-1β (Ahn et al., 2013). In addition, MSCs can also secrete growth factors and anti-inflammatory cytokines (Åkerud et al., 2001; Makar et al., 2008; Horie et al., 2011), thereby promoting a shift from a pro-inflammatory to an anti-inflammatory environment. In this case, the prevention of PHH was due to the anti-inflammatory effects of MSCs more than to their regenerative capacity (Ahn et al., 2013). In a subsequent study, this therapy was shown to be much more efficient when applied early in the animal’s inflammatory response to GMH/IVH (Park et al., 2016). Similarly, MSC potential as anti-inflammatory tool has been proven in mice with severe congenital hydrocephalus. Transplanted MSCs integrated between the reactive astrocytes and the damaged periventricular zones caused by the severe hydrocephalus. The tissue showed signs of reduction in apoptosis and in the levels of metabolites associated with hydrocephalus (García-Bonilla et al., 2020; Figure 11).

Unfortunately, transplanted MSC have not yet been found effective at repair the ependyma nor recovering neurogenesis (Ahn et al., 2013; Guerra et al., 2017). It has been proposed that grafting of neural stem cells into the brain of hydrocephalic fetuses would result in the repopulation of the neurogenic disrupted areas or the generation of a protective microenvironment to diminish/prevent the outcomes of neurogenic disruption, namely, hydrocephalus and abnormal neurogenesis (Guerra et al., 2017). In animal models with congenital hydrocephalus, without GMH/IVH, neural stem cells have been transplanted and neuroblasts were observed to differentiate from those cells (Henzi et al., 2020). The efficiency of this type of neural stem cell-based therapy in an inflammatory environment generated by GMH/IVH has not been yet reported. In the same direction, no stem cell-based therapy in an inflammatory environment generated by GMH/IVH with efficient recovery of ependyma has been yet reported (Figure 11).

## 12. Conclusion

Neuroinflammatory processes, such as those induced by GMH/IVH and PHH in premature infants or neonates, damage the ependyma, alters normal neurogenesis and, if unaddressed, causes neurological defects that may become chronic. In this cascade of events, ependyma has a main role. Bleeding in the germinal matrix, generates an inflammatory reaction in the tissue, and de-differentiation of the multiciliated ependymal cells by degradation of Foxj1 transcription factor and disruption of Ank3. De-differentiation of the multiciliated ependymal cells also is characterized by loss of mature cell adhesion and loss of mature paracrine signaling, and therefore dismantles the ependymal control over stem cells. Over time, de-differentiation of the multiciliated ependymal leads to de-structuration of ependyma epithelium, and proliferation of periventricular astrocytes in the periventricular astroglia reaction is induced. The astroglia reaction further disrupts ependyma structure where instead the astrocytes become proliferative.

Ependyma is also a key structure for the maintenance of the normal homeostasis of the brain. An abnormal CSF flow due to ependymal alteration leads to altered water transport, CSF circulation and filtering toward the brain parenchyma. This contributes to the development of PHH and the increase of the damage in the ventricular neurogenic region.

In this way, ependyma damage is a key element that needs to be addressed when trying to recover normal brain development after neuroinflammation, specially neuroinflammation triggered by GMH/IVH.

The conventional surgical therapies to treat PHH are based on draining and diverting the CSF and are not directed to recover the ependyma after its damage. In the same way, the stem cell-based strategies to treat GMH/IVH, only address the inflammatory reaction.

Removing blood with classical surgical approaches or reducing tissue inflammation by MSC therapies does help to reduce the first impact of the hemorrhage and reduce the inflammatory cascade of events that is triggered in the absence of treatment. However, the damaged ependyma cannot be recovered and therefore neurogenesis will be defective.

The development of new therapies aimed specifically to assess the recovery of the ependyma is necessary to restore homeostasis of the brain, maintain postnatal neurogenesis and reduce the functional damage in the normal brain development of the neonate. It may be that combinatory effects of different stem cell types could be a reasonable therapeutic approach to recover the ependymal function and, therefore, neurogenesis while also reducing the inflammatory response. Specific stem cells directed to recover Foxj1+ Ank3+ ependymal cells and repopulate the ventricular wall, and/or specific stem cells directed to recover the missing neural stem cells, are likely a good strategy. As there are no clinical results in ependymal recovery therapy yet, this important gap in the treatment of GMH/IVH and PHH persists.

## Author contributions

PP-G contributed to conception and design of the review and wrote the final version of the manuscript. AJ, JL-d-S-S, LR-P, PP-G, and RC-F wrote sections of the manuscript. JL-d-S-S designed the figures. All authors contributed to manuscript revision, read, and approved the submitted version.

## Funding

The present work was supported by grant PI19/00778 (to AJ and PP-G) from the Instituto de Salud Carlos III, Spain, cofinanced by FEDER funds from the European Union. RYC-2014-16980 to PP-G from the Ministerio de Economía y Competitividad, Spain; UMA18-FEDERJA-277 from Plan Operativo FEDER Andalucía 2014–2020 and Universidad de Málaga to PP-G; Contrato Postdoctoral-PPITD-UMA from Universidad de Málaga to LR-P; the Waite Student Bursary Prize from Society for Research Hydrocephalus and Spina Bifida to JL-d-S-S, and Proyectos dirigidos por jóvenes investigadores from Universidad de Málaga to PP-G.

## Conflict of interest

The authors declare that the research was conducted in the absence of any commercial or financial relationships that could be construed as a potential conflict of interest.

## Publisher’s note

All claims expressed in this article are solely those of the authors and do not necessarily represent those of their affiliated organizations, or those of the publisher, the editors and the reviewers. Any product that may be evaluated in this article, or claim that may be made by its manufacturer, is not guaranteed or endorsed by the publisher.
